# Evaluating Complications, Success Rate, and Marginal Bone Loss in Cement- Versus Screw-Retained Single Implant-Supported Zirconia Crowns: A Systematic Review and Meta-Analysis

**DOI:** 10.7759/cureus.83370

**Published:** 2025-05-02

**Authors:** Sidhartha Tomar, Deepesh Saxena, Pragati Rawat, Sukhmeen Madaan, Ayush Kumar

**Affiliations:** 1 Prosthodontics and Crown &amp; Bridge, Subharti Dental College and Hospital, Swami Vivekanand Subharti University, Meerut, IND; 2 Dentistry, Tri-Town Dental Clinic, Haileybury, CAN

**Keywords:** cement-retained crown, marginal bone loss, screw-retained crown, survival rate, zirconia prosthesis

## Abstract

This systematic review and meta-analysis aimed to compare marginal bone loss (MBL), bleeding on probing (BOP), and the incidence of complications between cement-retained and screw-retained (SR) single implant-supported zirconia crowns to provide evidence-based guidance for clinicians. A comprehensive search of PubMed/MEDLINE, Scopus, Embase, Google Scholar, and Lilac databases was conducted by two independent reviewers for studies published between January 2000 and March 2024. Studies evaluating the success rates and complications of single zirconia implant crowns with either cement or screw retention were included. Data on complication frequency, types, and overall success rates were extracted and analyzed. A total of 168 cement-retained and 166 SR zirconia crowns from seven studies with follow-ups ranging from 3 to 10 years were included. Meta-analysis of three to six studies per outcome indicated that SR crowns had significantly lower MBL at six months (standardized mean difference (SMD): -0.63; p = 0.002), though no significant differences in MBL were found at one, three, and five years. BOP outcomes were similar between both retention types. Notably, SR restorations demonstrated a 46% reduced risk of complications compared to cement-retained ones (RR: 0.54; p = 0.02). In conclusion, SR implant-supported zirconia crowns are associated with fewer early MBL and complications. However, long-term bone loss and peri-implant mucosal health appear comparable between the two retention methods.

## Introduction and background

With excellent long-term clinical results, implant-supported single crowns have become a reliable option for replacing a single tooth [1-4]. While metal abutments are traditionally considered the gold standard, providing exceptional survival rates across all jaw areas, their use in aesthetic regions can compromise treatment aesthetics [5-7]. All-ceramic crowns offer a solution by enhancing aesthetics, particularly in areas with thin mucosa. Zirconia abutments, known for their favorable biocompatibility with both hard and soft tissues, hold promise for addressing these aesthetic concerns [7-12].

Even though zirconia-based restorations supported by implants have great clinical survival rates, resembling those of tooth-borne reconstructions, they are susceptible to chipping, a challenge attributed to the absence of periodontal receptors [13-19]. The decision between cement-retained and screw-retained (SR) crowns remains crucial. While clinical studies have reported comparable results for both options, cemented restorations are associated with more severe biological complications, potentially due to residual excess cement [20-22]. Conversely, SR prostheses, although removable, are prone to technical issues [23-26].

According to systematic evaluations, SR reconstructions had a higher frequency of technical complications, while cemented crowns are associated with more biological complications [27,28]. A preclinical investigation demonstrated no variations in inflammatory infiltrates surrounding cement-retained crowns compared to SR crowns [29].

The optimum choice among cemented- and SR implant crowns on zirconia abutments is ambiguous regarding the yields of superior clinical and biological results. When all-ceramic crowns with personalized zirconia abutments are cemented, they demonstrate excellent long-term clinical results and stable marginal bone levels; however, data on SR crowns on zirconia abutments are limited [30-35].

Recent CAD/CAM (computer-aided design/computer-aided manufacturing) technologies provide SR and cemented all-ceramic reconstructions with custom-made zirconia abutments, providing clinicians with versatile options for implant-borne restorations. However, careful consideration of potential complications associated with each retention method is essential. Cemented crowns might cause fewer technical challenges; however, they have been associated with a higher occurrence of biological complications. Conversely, SR reconstructions offer easier retrievability but are more prone to technical issues. Clinicians must evaluate these parameters to determine the most appropriate retention method according to individual patient requirements and clinical conditions [36].

A comprehensive systematic evaluation comparing SR and cement-retained single implant-supported zirconia crowns is absent, despite the vast literature on the subject. As a result, current research hypothesizes that cement- versus SR zirconia implant crowns differ significantly concerning marginal bone loss (MBL), success rate, and complications.

## Review

Materials and methods

This systematic review protocol has been registered under registration number CRD 42024545371 in the National Institute of Health Sciences' International Prospective Register of Systematic Reviews (PROSPERO) database. The PRISMA (Preferred Reporting Items for Systematic Reviews and Meta-Analyses) standards were followed when retrieving information [37,38].

Focused Question

The focused question was: "What are the clinical outcomes, complications, marginal bone loss, and success rates of cemented versus screw-retained zirconia single implant-supported crowns?"

PICO Question

The PICO (population, intervention, comparison, and outcome measures) question is given in Table 1.

**Table 1:** PICO question (population, intervention, comparison, and outcome measures)

Population	Patients who received single implant-supported zirconia crown
Intervention	Zirconia crown
Comparison	Screw- vs. cement-retained zirconia crown
Primary Outcome	Marginal bone loss, clinical complication, and success rate
Secondary Outcome	Bleeding on probing

Eligibility Criteria

Human randomized clinical trials (RCTs) or controlled clinical trials that have been published in English between January 2000 and March 2024, including two treatment modalities namely cement-retained zirconia crown and single implant-supported screw, were required to fulfill inclusion criteria. Additionally, consecutive research had to have at least 10 dental implant-supported crowns for each group and a mean follow-up period of three years or longer.

Publications in languages other than English, case reports, case series, investigations with a follow-up of less than three years, educational announcements, expert opinions, narrative reviews, animal studies, and in vitro experiments were among the exclusion criteria.

Literature Search Protocol

Following established inclusion and exclusion criteria, two reviewers (ST and DS) independently conducted the search, evaluated publications, and compiled a list of their abstracts for inclusion. After comparing the reviewer’s lists of chosen articles, a kappa (κ) value was computed to determine the degree of agreement. Following a thorough examination of all the articles, a discussion was held to determine conclusions regarding inclusion [39].

Online databases such as Embase, PubMed/MEDLINE, Scopus, Lilac, and Google Scholar were employed for systematic literature searches. Keywords and search term combinations employed in the medical subject heading (MeSH) included "zirconia," "zirconia crown," "cemented," "cemented-retained," "implant-supported," "screw-retained," "marginal bone loss," "crestal bone loss," and "single crown." Boolean operators and wildcards will be used to ensure a thorough search.

Literature Selection and Data Extraction Protocol

Two reviewers (PR and ST) extracted the data. The general research features (authors, year of publication, kind of study, number of participants, and prosthesis) and implant features (quantity, placement, kind of prosthetic loading of implants, and follow-up time after implantation) along with primary and secondary results were used to extract relevant data (Table 2) [37-43].

**Table 2:** Characteristics features of included studies RCT: Randomized control trial; CR: Cemented reconstruction; SR: Screw-retained; n: Sample size; mm: Millimeters; CEM: Cement; MBL: Marginal bone loss; ZrC: Zirconia crown; FCA: Full crown abutment.

Authors	Study design	Sample size	Follow-up	MBL	Results	Conclusions
Kraus et al., 2019 [37]	RCT	Twenty cemented reconstruction (CR) and 20 screw-retained (SR) ceramic single crowns on two-piece dental implants were randomly assigned to 44 patients.	3 years	Within groups SR and CR, the mean MBL at 3 years were −0.4 mm (−0.5; −0.3) and −0.4 mm (−0.6; −0.3), respectively (P = 0.864).	Results related to biological, technological, and radiographic outcomes did not substantially differ between the groups (P > 0.05). In the case of the CR group, one implant (2.3%) was lost. The peri-implant disease necessitated the removal of one more cemented crown (2.3%). Due to a zirconia abutment fracture, six patients (13.6%) lost their reconstructions (4 SR and 2 CR).	After three years, the overall survival outcomes (technical, biological, and radiological) of CR and SR were comparable. Technical issues occurred often in both groups.
Heierle et al., 2019 [42]	RCT	An SR crown with a directly veneered zirconia abutment (SCREW) or a cemented lithium disilicate crown on a tailored zirconia abutment (CEM) was used to restore 34 patients with single-tooth implants at random. Marginal bone level and technical parameters were evaluated at baseline and three years post-loading.	3 years	There were no statistically significant differences between the baseline and the three-year follow-up (P = 0.70) or at three years (P = 0.20) according to intergroup comparisons.	The median differences were -0.1 mm (CEM; intragroup P = 0.36) and -0.0 mm (SCREW; intragroup P = 0.58) between the baseline and the three-year follow-up. When comparing groups, statistically significant differences were not seen at three years (P = 0.20) or throughout time (P = 0.70).	After three years, the radiographic and technical results of SR and cemented reconstructions were almost identical.
Amorfini et al., 2018 [43]	RCT	In the anterior regions, 32 patients underwent single-tooth restorations supported by implants that were fitted with zirconia abutments.	10 years	There was no discernible change in MBL between the ZrC and FCA groups, measuring 0.82 and 0.95 mm, respectively.	During the course of the trial, there were no implant failures, and 94% of crowns remained functional after 10 years. Both groups (3 FCA and 2 ZrC) had prosthetic problems documented; however, there was no discernible difference (P = 0.65).	There was no discernible difference between the groups' MBLs, which were 0.82 mm in the FCA group and 0.95 mm in the ZrC group. It is necessary to validate these positive first findings with longer-term monitoring of bigger study samples.
Cacaci et al., 2017 [38]	RCT	This randomized prospective trial included 58 individuals with 114 implants. On the implant, zirconia-based crowns with a sintered veneering cover were either cemented (n = 61) or SR (n = 53).	3 years	Not given	There were no implant failures or crown fractures. About 1.8% of the cases had veneering porcelain fractures after an average clinical service period of 36.9 months.	During the limited mean observation duration of 36.9 months, implant-supported zirconia-based crown copings veneered with a high-strength ceramic by sintering, both cement-retained and SR, demonstrated an acceptable success rate under clinical settings for premolar and molar regions.
Lamperti et al., 2022 [39]	RCT	An SR crown or a cemented lithium disilicate crown on a single-piece customized zirconia abutment (CEM, 17 patients) was used to repair 34 patients with single-tooth implants at random.	5 years	After 5 years, the median marginal bone level was found below the implant shoulder at −0.15 mm (IQR: −0.89; 0.27) for CEM and −0.26 mm (IQR: −0.38; 0.01) for SCREW.	Both groups experienced 100% and 82.4% survival rates on the implant and restorative levels, respectively. About 15.4% (CEM) and 15.4% (SCREW) of all technical complications were reported after five years (intergroup p = 1.00). Over time, clinical parameters (baseline to 5 years) stayed consistent.	The clinical, technical, and radiological results of SR and cemented restorations were almost identical after five years. Both groups experienced regular technical issues.
Kraus et al., 2022 [40]	RCT	In the esthetic zone, 44 single-tooth gaps were filled with 44 two-piece dental implants. Patients underwent a single-crown all-ceramic SR or cemented (CR) at random.	5 years	The implant shoulder was situated slightly apically, with the median MBL measuring 0.4 mm (0.5; 0.3) in the SR and 0.4 mm (0.8; 0.3) in the CR.	Both the biological complication rate (36.8%, SR: 0.0%; intergroup p = 0.0022) and the overall complication rate (68.4%, SR: 22.7%, intergroup p = 0.0049) were considerably greater in cemented restorations. Both of the groups did not substantially vary in any other outcomes (p > 0.05).	The survival rate of single-tooth restorations made entirely of ceramic on two-piece dental implants was comparatively poor. Compared to SR restorations, cemented restorations were linked to a higher prevalence of biological and general complications.
Thoma et al., 2018 [41]	RCT	A cemented lithium disilicate crown on a customized zirconia abutment (CEM) or an SR crown with a directly veneered zirconia abutment (SCREW) was randomly assigned to 33 patients who had single-tooth implants.	5 years	At crown placement, the group CEM had median MBL of 0.31 mm (Q1 = 0.13; Q3 = 0.83), while the group SCREW had median MBL of 0.47 mm (Q1 = 0.25; Q3 = 0.70). The MBL was measured at 0.32 mm (Q1 = 0.12; Q3 = 0.87) (CEM) and 0.36 mm (Q1 = 0.21; Q3 = 0.61, SCREW) during the six-month follow-up.	After six months, the implant and crown survival rates were 100% in the 33 patients who finished the research. Histologically, group CEM tended to have more inflammatory cells overall (p > 0.05). Furthermore, in comparison to the junctional epithelium and supracrestal connective tissue, the sulcular epithelium contained notably fewer inflammatory cells and fibroblasts/fibrocytes (p < 0.001).	More inflammatory cells have been related to cement reconstructions, and periodontopathogen diagnoses were made in a greater number of patients. Similar radiographic results (MBL) and clinical outcomes (bleeding on probing and probing depth) were obtained with both types of reconstructions.

Risk of Bias

Two investigators (ST and DS) independently assessed the risk of bias by employing the Cochrane Risk of Bias Tool v2.0 (RoB 2) for randomized trials. The following criteria have been the focus of this tool: inadequate outcome data (attrition bias), selective reporting (reporting bias), blinding of outcome assessment (detection bias), blinding of participants and personnel (performance bias), and further potential causes of bias. In case of any disagreement, a third author (PR) was consulted to settle disagreements.

Statistical Analysis

Review Manager v5.4 (RevMan; Nordic Cochrane Centre, Copenhagen) was utilized to conduct meta-analyses by applying the random effects model. The Q-test was employed for evaluating heterogeneity, and I^2^ statistics were employed to quantify it. Data on MBL, bleeding on probing (BOP), and complications were obtained from the selected studies. The random effects model was employed for analyses if the test revealed significant heterogeneity (I^2 ^> 50%), while the fixed effects model was employed if I^2^ ≤ 50%.

Results

Study Selection

**Figure 1:**
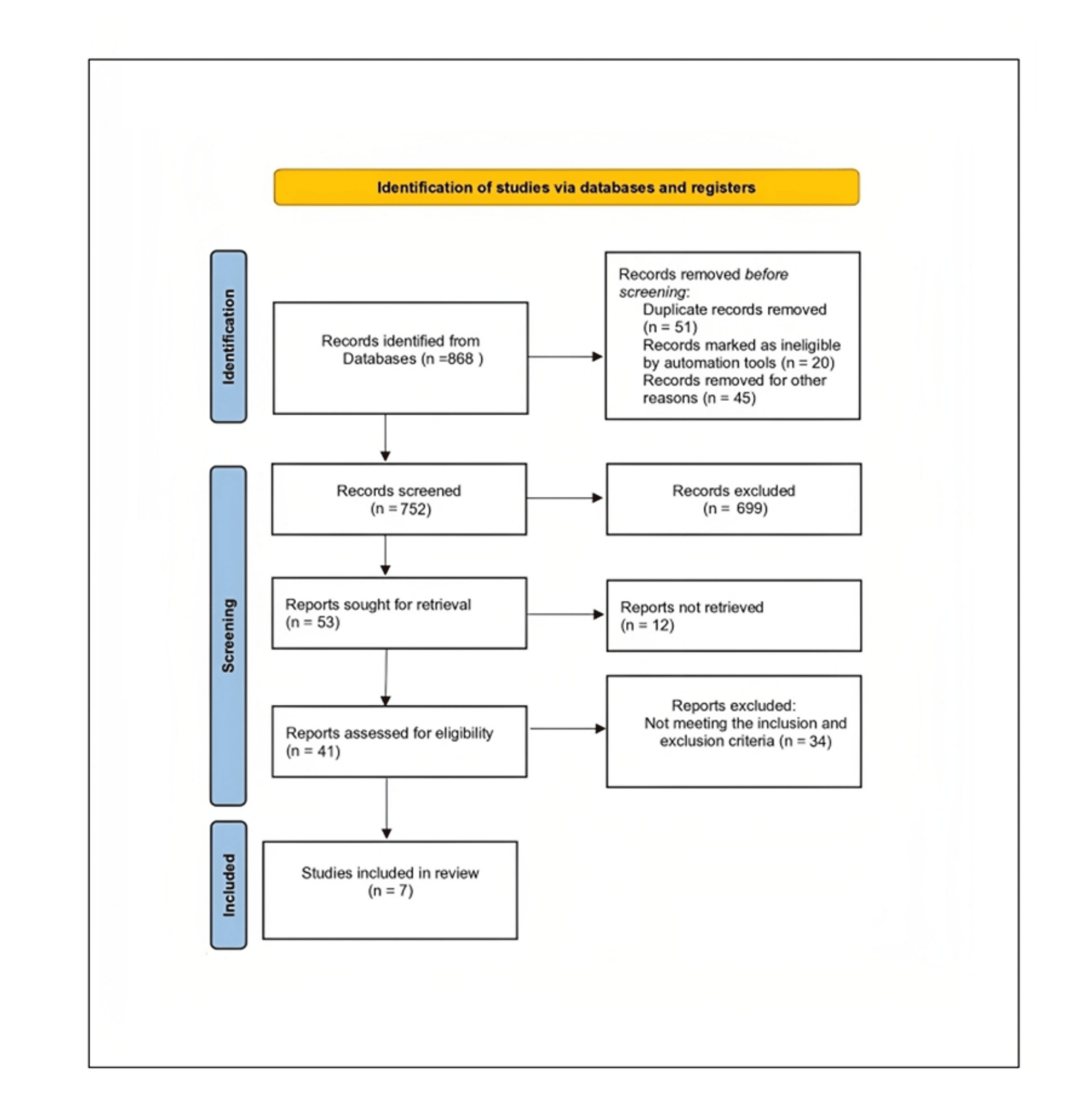
PRISMA (Preferred Reporting Items for Systematic Reviews and Meta-Analysis) flow diagram This figure details the disposition of screened, included, and excluded records.

Initial electronic literature searchesin Google Scholar, Scopus, PubMed/MEDLINE, and Lilac databases identified 868 titles (Figure 1). A total of 817 publications went through screening following formal inclusion and exclusion criteria, after duplicate studies had been eliminated. Fifty-three abstracts were chosen for additional analysis after being evaluated against the inclusion criteria. Of these, 34 publications were given full-text consideration, and 22 papers were first included for additional analysis after full-text analysis. Seven RCTs were ultimately determined to be relevant for qualitative synthesis and systematic review.

Risk of Bias Assessment

**Figure 2:**
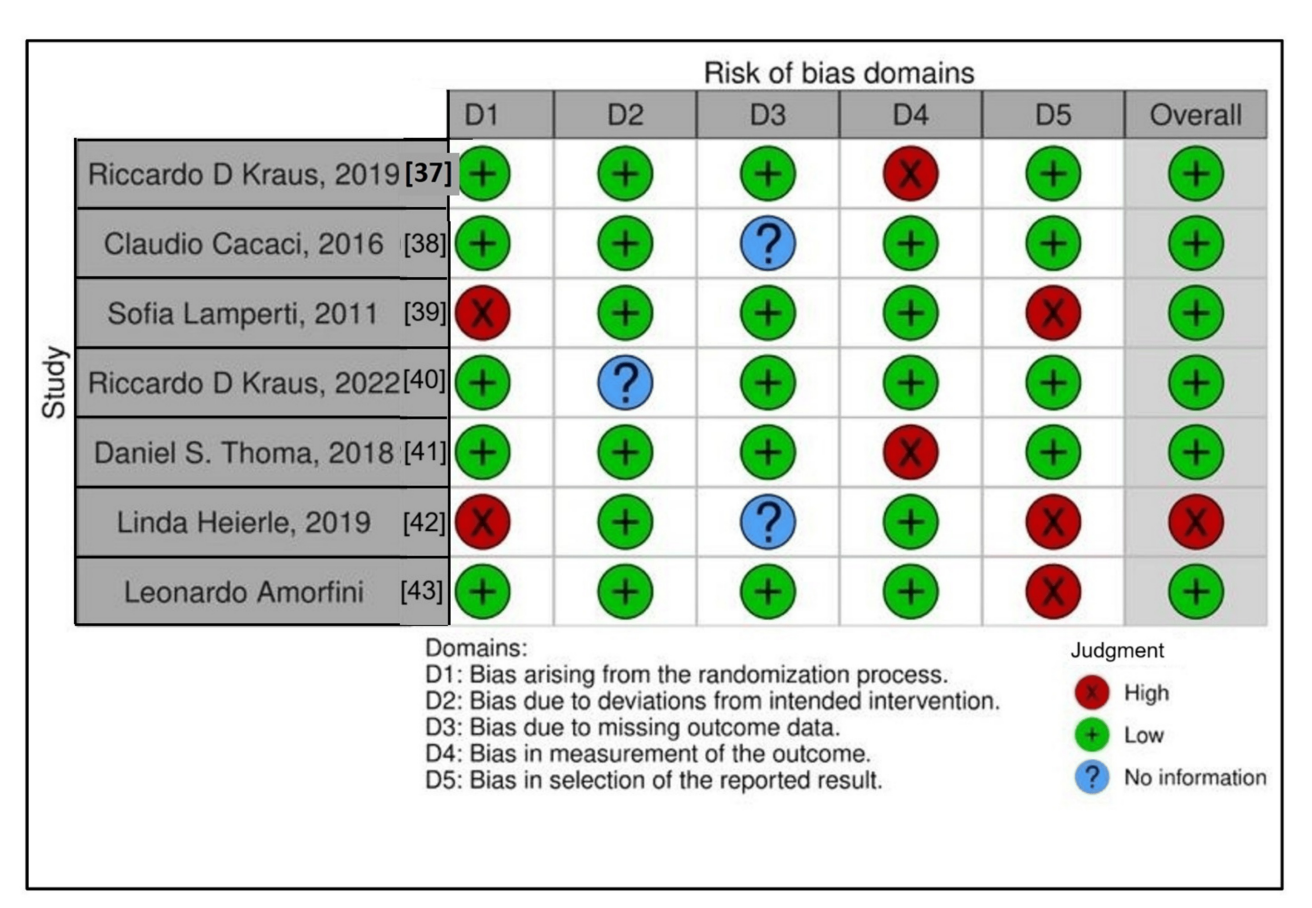
Risk of bias of selected studies A low risk of bias has been identified in six investigations.

A low risk of bias was identified in six investigations. Three research investigations indicated no evidence of bias in any of the three important domains; two research reported a high risk of bias in only one important domain; and three additional investigations indicated a high risk of bias in one of the important domains (Figure 2) [37-43].

Meta-analysis

Comparison of the MBL After Six Months

**Figure 3:**
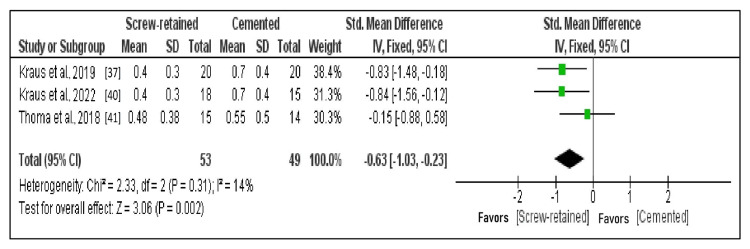
Forest plot of MBL after six months Three studies that fulfilled the criteria for data outcomes that could be subjected to quantitative analysis were the subjects of the meta-analysis. A standardized mean difference between screw-retained and cemented implants was -0.63 (95% CI: -1.03 to -0.23; p = 0.002), indicating a significant decrease in the mean MBL. MBL: Marginal bone loss.

Three studies that fulfilled the criteria for data outcomes that could be subjected to quantitative analysis were the subjects of meta-analysis. A forest plot represents the overall comparison results (Figure 3) [4,37,40]. A fixed effects model was employed, considering heterogeneity in the meta-analysis of selected research was >50% (I^2 ^= 14%). The standardized mean difference between SR and cemented implants was -0.63 (95% CI: -1.03 to -0.23; p = 0.002), indicating a significant decrease in mean MBL.

Comparison of the MBL After One Year

**Figure 4:**
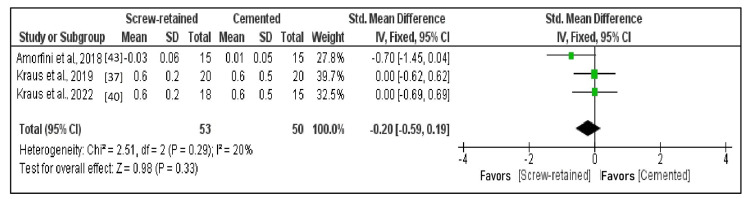
Forest plot of MBL after one year With a standardized mean difference of -0.20 (95% CI: -0.59 to 0.19), the mean MBL had been lower in screw-retained implants than in cemented implants. MBL: Marginal bone loss.

Three studies that fulfilled requirements for data results that could be subjected to quantitative analysis constituted part of the meta-analysis. The forest plot represents the overall comparison results (Figure 4) [37,40,43]. A fixed effects model was employed since heterogeneity in the meta-analysis of selected research was less than 50% (I^2 ^= 20%). With a standardized mean difference of -0.20 (95% CI: -0.59 to 0.19), the mean MBL was lower in SR implants than in cemented implants; however, there was an insignificant difference in marginal bone loss among the two methods after a year (p = 0.33).

Comparison of the MBL After Three Years

**Figure 5:**
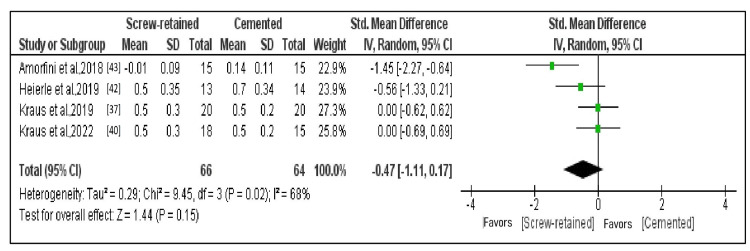
Forest plot of MBL after three years With a standardized mean difference of -0.47 (95% CI: -1.11 to 0.17), the mean MBL was lower in screw-retained implants than in cemented implants. MBL: Marginal bone loss.

Four research investigations that fulfilled requirements for data outcomes that could be subjected to quantitative analysis constituted subjects of the meta-analysis. The forest plot represents the overall comparison results (Figure 5) [37,40,42,43]. A random effects model was employed since heterogeneity in the meta-analysis of certain selected research was greater than 50% (I^2 ^= 68%). With a standardized mean difference of -0.47 (95% CI: -1.11 to 0.17), the mean MBL was lower in SR implants than in cemented implants; however, there was an insignificant difference in marginal bone loss among the two methods after three years (p = 0.15).

Comparison of the MBL After Five Years

**Figure 6:**
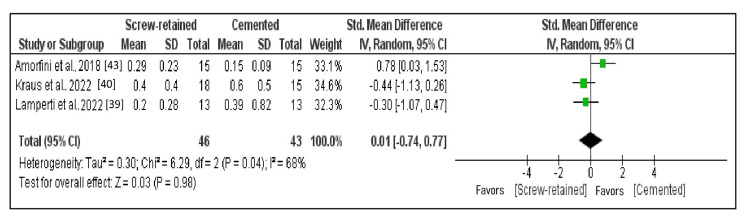
Forest plot of MBL after five years At a standardized mean difference of 0.01 (95% CI: -0.74 to 0.77), the mean MBL for screw-retained and cemented implants was nearly identical, and the difference in marginal bone loss after five years between the two techniques was not statistically significant (p = 0.98). MBL: Marginal bone loss.

The meta-analysis was conducted on three studies that fulfilled the criteria for having data outcomes that could be subjected to quantitative analysis. Total comparison results were represented as forest plots (Figure 6) [39,40,43]. The random effects model was employed since the meta-analysis of several selected research studies revealed heterogeneity of greater than 50% (I^2 ^= 68%). At a standardized mean difference of 0.01 (95% CI: -0.74 to 0.77), the mean MBL for SR and cemented implants was nearly identical, and the difference in marginal bone loss after five years between the two techniques was not statistically significant (p = 0.98).

Comparison of the Bleeding on Probing

**Figure 7:**
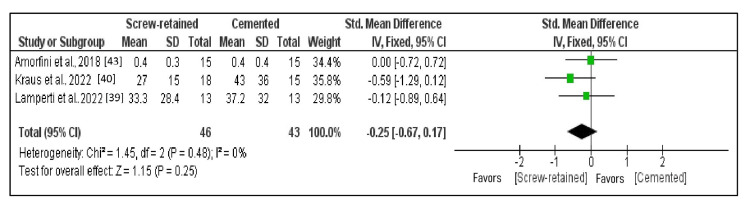
Forest plot of BOP The mean BOP score is almost similar in screw-retained implants and cemented implants with a standardized mean difference of 0.01 (95% CI: -0.67 to 0.17). BOP: Bleeding on probing.

Three studies that fulfilled requirements for data outcomes that could be subjected to quantitative analysis constituted subjects of the meta-analysis. The forest plot represents the overall comparison results (Figure 7) [39,40,43]. With the meta-analysis conducted for selected studies, heterogeneity was less than 50% (I^2 ^= 0%); hence, a fixed effects model was applied. The mean BOP score had been almost similar in SR implants and cemented implants with a standardized mean difference of 0.01 (95% CI: -0.67 to 0.17), and the difference in the bleeding on probing between the two methods was nonsignificant (p = 0.25).

Comparison of the Complications

**Figure 8:**
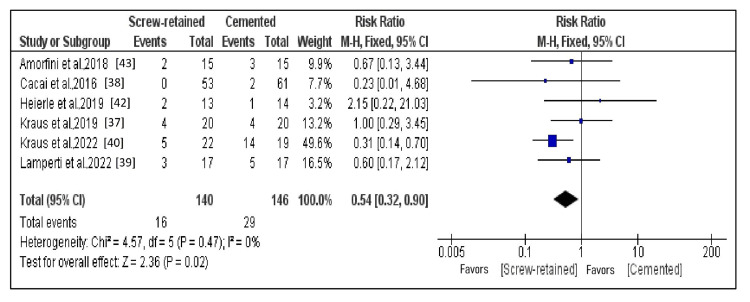
Forest plot of complications With a risk ratio of 0.54 (95% CI: -0.32 to 0.90), screw-retained implants had nearly 46% lower complications (minimum duration of assessment: three years) than cemented implants, and the difference in complications between the two groups appeared significant (p = 0.02).

Six studies that fulfilled the criteria for data outcomes that could be subjected to quantitative analysis constituted subjects of the meta-analysis. A forest plot represents the overall comparison results (Figure 8) [37-40,43,44]. A fixed effects model was employed, as the heterogeneity in the meta-analysis of the selected studies was less than 50% (I^2^ = 0%). With a risk ratio of 0.54 (95% CI: -0.32 to 0.90), SR implants had nearly 46% lower complications (minimum duration of assessment: three years) than cemented implants, and the difference in complications between the two groups appeared significant (p = 0.02).

Discussion

This systematic review utilized the Cochrane Collaboration methods and the latest PRISMA criteria to evaluate the best evidence on the success rate and complications of cement- or SR single implant-supported zirconia crown. The results of selected RCTs do not support the hypothesis that there is a significant difference in the success rate and clinical complications of screw- and cement-retained single implant-supported zirconia crowns. Instead, they demonstrate no significant difference in these results.

Depending on the extent of reconstructions, this systematic literature review revealed significant differences in the performance of SR versus cemented implant reconstructions. However, when comparing cemented and SR crowns, there were no differences in the expected five-year survival rates of implants supporting single crowns.

Frequently, SR reconstructions encountered more technical complications than cemented ones. More specifically, compared to cemented reconstructions, SR reconstructions exhibited greater rates of veneering ceramic chipping and abutment/reconstruction screw loosening. On the contrary, cemented reconstructions had a greater rate of severe biological complications, especially bone loss. It became apparent that an excess of cement had been the cause of this problem [44,45]. Alternatively, it had been stated that loose abutments were more often associated with this condition [46].

However, the types of complications associated with each retention method differ, with SR crowns exhibiting a higher incidence of technical issues and cement-retained crowns showing a higher incidence of biological complications. Neither of the two types of fixation demonstrated a clear advantage over the other.

A prior systematic review by Sailer et al. investigated SR versus cement-retained prostheses, reporting both types of reconstructions influenced clinical outcomes in different ways, with neither fixation method demonstrating a clear advantage over the other [44]. However, for extensive implant reconstructions, such as partial or full-arch fixed dental prostheses (FDPs), SR designs are recommended. To our understanding, there is not a systematic review that compares cement-retained with SR zirconia single implant-supported crowns. 

This systematic review's scope has been restricted in several ways. First, publication bias probably happened as a result of limitations to English-language publications, which might have led to the omission of significant research. The results might additionally have been affected by differences in the included studies' clinical procedures, patient populations, and research designs. Despite these drawbacks, the general patterns identified in this analysis offer valuable insights into the efficacy and difficulties of various fixation methods.

## Conclusions

This meta-analysis underscores the clinical advantages of SR implants, particularly in the short term, where they exhibit significantly lower MBL and fewer complications compared to cemented implants. The substantial decrease in complications with SR implants indicates a favorable long-term prognosis, even though the difference in MBL between the two implant types diminishes over longer periods. Moreover, both implant types maintain similar peri-implant mucosal health as indicated by comparable BOP scores.

Given these findings, SR implants may be preferred, especially in cases where retrievability and long-term maintenance are of concern. However, the choice between SR and cemented implants should also consider individual patient factors, clinician preference, and specific clinical scenarios. It is recommended that further extensive research be conducted employing standardized procedures to validate these results and offer more conclusive recommendations for clinical practice.
